# Integrating Moving Platforms in a SLAM Agorithm for Pedestrian Navigation

**DOI:** 10.3390/s18124367

**Published:** 2018-12-10

**Authors:** Susanna Kaiser, Christopher Lang

**Affiliations:** German Aerospace Center (DLR); Institute for Communications and Navigation, Oberpfaffenhofen, 82234 Wessling, Germany; christopher.lang@tum.de

**Keywords:** indoor navigation, pedestrian dead reckoning, simultaneous localization and mapping, FootSLAM, moving platform detection

## Abstract

In 3D pedestrian indoor navigation applications, position estimation based on inertial measurement units (IMUss) fails when moving platforms (MPs), such as escalators and elevators, are not properly implemented. In this work, we integrate the MPs in an upper 3D-simultaneous localization and mapping (SLAM) algorithm which is cascaded to the pedestrian dead-reckoning (PDR) technique. The step and heading measurements resulting from the PDR are fed to the SLAM that additionally estimates a map of the environment during the walk in order to reduce the remaining drift. For integrating MPs, we present a new proposal function for the particle filter implementation of the SLAM to account for the presence of MPs. In addition, a new weighting function for features such as escalators and elevators is developed and the features are learned and stored in the learned map. With this, locations of MPs are favored when revisiting the MPs again. The results show that the mean height error is about 0.1 m and the mean position error is less than 1 m for walks with long distances along the floors, even when using multiple floor level changes with different numbers of floors in a multistory environment. For walks with short walking distances and many floor level changes, the mean height error can be higher (about 0.5 m). The final floor number is in all cases except one correctly estimated.

## 1. Introduction

In order to solve the 3D indoor pedestrian navigation problem, there exist various approaches. Infrastructure based solutions usually apply additional transmitters or receivers as it is the case when using, e.g., ultra wideband (UWB), iBeacons, radio frequency identification (RFID) or global navigation satellite system (GNSS) repeaters. An overview of indoor navigation solutions can be found in [1,2,3,4]. A promising infrastructure-free solution for navigation in GNSS-denied areas such as indoors, tunnels, urban canyons or even mines is pedestrian navigation solely based on inertial sensor units. They only collect data from the agent itself, and therefore are no subject of privacy concerns such as vision. The fact that they operate without pre-installed infrastructure makes them suitable for special applications such as fire fighters rescuing, policemen supervision, industrial inspections, or monitoring of elderly people. For instance, in the case of a fire, it would be desirable for the fire station to know the state and position of an injured fire fighter in operation in order to rescue him. In this case, cameras cannot be used due to the fact that fire fighters enter a smoky environment when there is a fire. The infrastructure might be destroyed by fire, so that fire fighters cannot rely on solutions based on signals of opportunities from pre-installed transmitters. Therefore, a solution solely based on inertial sensor units is advantageous.

PDR techniques provide the step and heading displacement estimations of the pedestrian’s walk either by applying a strapdown system [5,6] or a step and heading system (SHS) [7]. Strapdown systems can be used when the sensor is mounted on the foot whereas SHSs are applied when the sensor is mounted at other locations of the human body. However, a disadvantage of a PDR technique is that the trajectory is affected by a remaining drift (rather a heading drift than a drift in the step length). To reduce or correct the drift a SLAM algorithm [8,9] can be applied, which originally was developed for robots and where landmarks or features are learned. In the meantime, different kinds of SLAM algorithms have been investigated during the last decades. Examples among them are EKF-SLAM (extended Kalman filter SLAM) [8], FastSLAM [10], GraphSLAM [11], SemanticSLAM [12], SignalSLAM [13], ActionSLAM [14], or VisualSLAM [15]. In this work, we apply FootSLAM, a SLAM algorithm based on step and heading measurements of inertial sensors originally mounted on the foot, that was developed at the German Aerospace Center (DLR) [16,17]. FootSLAM can provide accurate positioning in many structured environments, where no GNSS signal is available. FootSLAM’s main principle is that walkable areas of the whole environment, instead of distributed landmarks, are learned during tracking a pedestrian online. The advantage of FootSLAM is that revisiting any area—not only specific landmarks—will lead to drift correction and that it is very robust applying inertial sensors only. This is of particular interest for professional use cases that are the focus of this work. The positioning of FootSLAM can further be enhanced by applying the learned FootSLAM map information as prior information for future walks.

FootSLAM itself is not restricted to use the step and heading information from the sensor mounted on the foot. Instead, it can be applied for any odometry provided by a PDR. For instance, FootSLAM was already successfully used with the sensor mounted in the pocket or at the upper leg (PocketSLAM [18]). In the following, we will further name the developed SLAM algorithm FootSLAM despite the fact that this is misleading to be restricted to sensors mounted on the foot. By using the original naming we want to distinguish between other SLAM algorithms and the FootSLAM method itself.

However, MPs such as escalators and elevators are not yet addressed inside the FootSLAM algorithm. The term MP denotes any (indoor) transportation platform, such as elevators, escalators, or moving sidewalks. In future pedestrian navigation applications (indoor and outdoor), the term MP can also include multimodal transportation such as driving a car or using public transport. In order to get the step and heading information from the inertial data, a PDR is applied where either the velocity is reset when the foot is still and stable, i.e., during stance phases of the gait (zero velocity update (ZUPT) [5], sensor mounted on the foot), or the steps are counted which are of much higher dynamics than the acceleration of an MP (SHS, sensor mounted at another location of the body). Unfortunately, with these constraints MPs would be ignored because constant velocity phases of the platform are overlooked. In this paper, we focus on the integration of MPs in the FootSLAM algorithm. After having detected the MP, the heading and velocity of the platform are estimated and with them the displacement due to the MP is estimated inside FootSLAM. In addition, the event of a floor transition via an MP is stored as a feature in the map in order to enhance the positioning and the time of convergence of the FootSLAM map. By doing so, special features such as elevators or escalators can be marked inside the FootSLAM map and the location of such environmental features can be learned during the walk. These scientific findings can also be adopted for multimodal transportation scenarios.

Beside using inertial sensors only, it is possible to fuse other sensor measurements or other information in the particle filter of FootSLAM like magnetometers (MagSLAM [19], Wireless Fidelity (WiFi) (WiSLAM [20]), marked places (PlaceSLAM [21])), GNSS, UWB, etc. In this work, we apply only the pure FootSLAM for showing results in infrastructure-free environments for professional applications such as fire fighters rescuing or policemen supervision, and for presenting the effects of the new proposed technique without having any influence of other fused signals.

The paper is organized as follows. In Section 1.1 an overview of the FootSLAM system is presented and in Section 1.2 the related work regarding moving platform detection (MPD) and handling MPs in a SLAM-algorithm is described. The Bayesian derivation of the FootSLAM system including features is introduced in Section 2. For including MPs in FootSLAM, a reliable MPD algorithm is needed. The MPD algorithm applied in this paper will be examined in Section 2.3. Experimental results are presented in Section 3. Finally, Section 4 is devoted to conclusions and future work.

### 1.1. System Overview

The FootSLAM hierarchy consists of two cascaded estimators. First, the step length and heading information is calculated from the raw inertial measurements in an unscented Kalman filter (UKF) [6]. These odometry measurements obtained from the lower UKF are then fed to the upper FootSLAM algorithm. The FootSLAM algorithm builds on a Rao-Blackwellized particle filter (RBPF) [22], implementing the principle of FastSLAM factorization [10]. The FootSLAM algorithm represents a likelihood particle filter, where the proposal is based on the measurement likelihoods and the weighting is based on the movement model, which exploits probabilistic constraints in conformity with the environment. Each particle represents the estimated position of the pedestrian and stores its own map of the environment. FootSLAM’s particle map is based on a hexagonal prism grid for 3D environments and the hexagonal prism edge transitions are counted during the walk and stored in the map. Following the FootSLAM principle, particles that revisit similar transitions are rewarded. In 3D environments, transitions that lie on top of each other are preferred in order to handle buildings that naturally are of repetitive structure over various floors. With the introduction of hexagonal prisms, floor transitions via stairs are handled in the 3D-FootSLAM algorithm [23].

### 1.2. Related Work

For integrating MPs, a reliable MPD is needed. In this section, we will first recapitulate related work on MPD techniques and after that give an overview of the related work regarding handling the MP in an indoor navigation algorithm.

MPD is a decisive part in the area of activity recognition. For instance, in the so called SemanticSLAM algorithm [12] the escalators and elevators are detected via a binary decision tree, which was also proposed for classification by [24]. A state machine was used in [25]. The authors of [26] tracked the pattern of the elevator ride by integrating the z-acceleration. A combination of consulting the integrated z-acceleration, the disturbances of the earth magnetic field and the barometric signal was proposed in [27]. Another acceleration phase tracking based on the detection of the acceleration and deceleration peaks was proposed in [12,25,28], where in [25,28] the velocity is corrected during the constant velocity phase.

Beside this, machine learning approaches can be used to classify MPs. A dynamic Bayesian network is consulted with a sensor in [29], that is trained before classification. Different statistical, energy, and time- and frequency-domain features are investigated in [30,31,32]. Feature selection was based on the information gain, the correlation based feature selection, and the decision tree pruning in [31], and on the correlation based feature selection and ReliefF feature selection techniques in [32]. The decision tree classification was used in [30], where [31,32] used additionally decision tree, decision tables, naive Bayes, k-nearest neighbor, logistic regression and multilayer perceptron (MLP) classification algorithms. In [31], escalators and elevators are used in a static way, where the features are based on the three axes acceleration data measured with a smartphone held in the left or right hand in front of the body. The authors claim that only few features in combination with MLP are necessary to reach a good accuracy. In [32], the investigations were performed with respect to short response times. It turned out that statistical features of the accelerometer and magnetometer signals in combination with random forest and k-nearest neighbor performed best for fast response times. Finally, in [33] pose invariant features of the accelerometer signals are consulted in combination with statistical features of the barometer signal. This is specially designed for smartphones for allowing the detection of elevators independently of the sensor’s location and orientation.

In this paper, we used the detection technique of [28] because it provides a very good performance in terms of precision and recall. In addition, with this technique, the velocity of the MPs is provided. This is needed for the implementation in FootSLAM and it will not be provided directly by the machine learning approaches. The use of classifier algorithms as in [32] is foreseen for future work. The main focus of this work is the correction of the missing movement during MP rides in the FootSLAM algorithm and the declaration of MPs as features in order to learn the positions in a common map.

Handling MPs in PDR can only be found in few 3D indoor navigation systems using inertial sensors in the literature. In [25,34], the MP is detected and the ZUPTs are suspended. In the so called activity map matching (AMM), the PDR system is re-calibrated by matching activities to prior known locations. In [35], the PDR uses AMM in order to reduce the drift of the estimated position with the knowledge of vertical links like staircases and elevators in a map. The features are calculated based on the raw acceleration measurements and fused by a support vector machine (SVM). Similarly, in [36], AMM is used in combination with fuzzy associative classifiers. The authors of [37] provide a mismatch probability of AMM. In [24], a method was proposed that uses activities in order to match them to an indoor road network based on an indoor map. This network is used to help to reduce the drift in pedestrian indoor navigation. A hidden Markov model (HMM) is used as the map matching method in this case. The scheme matches turning at corners, turning around, stairs, elevators and escalators. Known landmark positions mainly based on stairs and escalator positions are used in [38]. In the GraphSLAM [33] approach, landmark positions are collected in advance via training of their WiFi visibility (landmarks: stairs and elevators). In [39], the authors distinguish between seed landmarks and organic landmarks. Seed landmarks like stairs and elevators force the users to behave in predictable ways which results in a signature of a sensor. Organic landmarks are signatures such as WiFi and magnetic signatures which are dependent on the environment. These landmarks are used to correct the path estimated by the PDR. In our approach, we do not use any known building layout or trained landmark position, instead of it we learn the landmarks or feature positions during the walk. In addition, we do not use WiFi positioning having in mind professional use cases like fire fighters rescuing and policemen supervision.

Learning landmarks’ positions during the walk including MPs can also be found in the literature in the so called SemanticSLAM algorithm [12] and in the 3D-ActionSLAM [14] algorithm. In the SemanticSLAM, seed landmarks (stairs, elevators) as well as organic landmarks (WiFi, magnetic and inertial sensor landmarks) are learned in order to correct the position when revisiting it. In 3D-ActionSLAM further activities such as sitting, standing, stair climbing as well as wall-constrained walking patterns (corners, corridors) are considered as landmarks. Both algorithms rely on the FastSLAM approach. In this paper, we integrated landmarks (referred to as features) in the FootSLAM algorithm. The novelty of this paper is the new proposal function in the particle filter framework for correcting the position in the case of MPs and the integration of features into the FootSLAM estimation procedure while storing them in a common map. The advantage of using the FootSLAM algorithm compared to other SLAM algorithms is that, alongside the feature map (FM), a map of walkable areas is learned during the walk. With the learned features we are able to mark the features in that map and use them to refine the estimated path. These additions to the FootSLAM algorithm are generally valid and not restricted to a sensor mounted on the foot.

## 2. Bayesian Derivation

### 2.1. Dynamic Bayesian Network Representation

**Figure 1:**
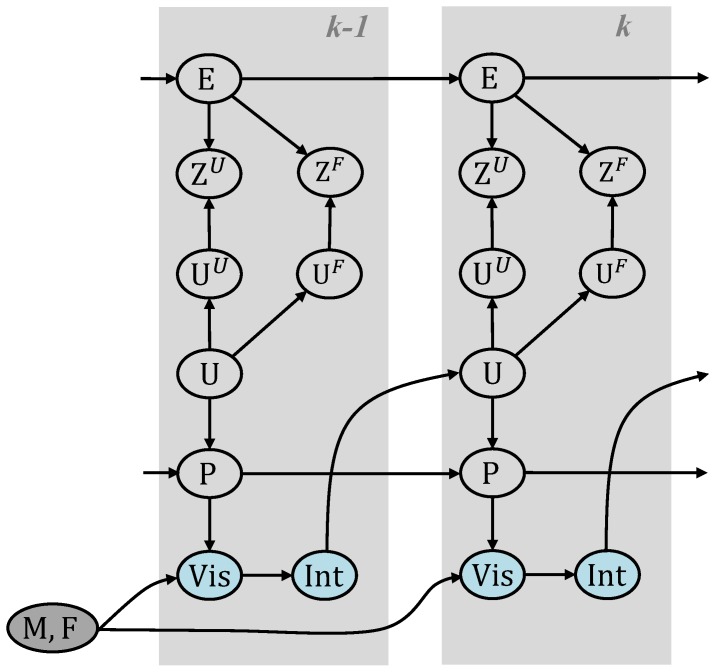
Extended dynamic Bayesian network (DBN) for FootSLAM with transportation platform support showing two time slices. M represents the environmental map, F the feature map, P the pose, U the odometry, Z the measurement random variable and E models the error. Environmental features influence the pedestrian’s visual impression Vis and intention Int. U is interpreted as a superimposed displacement from stepped locomotion $U^{U}$ and external platform locomotion $U^{F}$.

In order to derive the optimal Bayesian estimator, we formulate the positioning problem as a dynamic Bayesian network (DBN). We extend the FootSLAM DBN by a feature map based on our MP model. The resulting network is shown in Figure 1.

The pedestrian is considered as an integral part of the system, who navigates in a map M that embodies moving constraints through walls, doors, obstacles, and characteristic features like elevator and escalator position. The human relies on visual cues Vis to identify the environment and forms intentions Int where to go next. To reduce setup complexity, we employ no means to observe the human perception and intentions, instead we hitchhike on resulting steps $U_{k}$ at time *k* with odometry measurements $Z_{k}$, e.g., obtained by the ZUPT-aided inertial navigation system (INS) described in [6]. In addition, we learn the map of the environment that influences the human intentions and restricts the possible human motion. Since the map of walkable areas is learned during the walk, changes in the map will also be learned after a while. It is assumed that those changes happen only rarely.

In the real world, the odometry measurements are affected by measurement noise and correlated errors $E_{k}$. $P_{k}$ describes the pose, and changes in this position from time step k−1 to time *k* are represented by the pedestrian displacement vector $U_{k}=P_{k}-P_{k-1}$.

Additionally, there are specific features or landmarks, which guide the pedestrian when they intend to perform a certain location-related activity such as changing the floor in a multistory building. We propose to mark such locations through feature identifiers $F_{k}$ that will be stored in a FM F.

For incorporating MPs into this system, we interpret $U_{k}$ as superimposed displacements from stepped locomotion $U_{k}^{U}$ and external locomotion through MPs $U_{k}^{F}$. This simplification suggests that the platform adds a motion to the pedestrians without preventing them from stepping within the influence area of the MP. With an IMUs mounted on the foot, we can only reliably measure the continuous movement of the platform during stance phases, but we can detect its occurrence by detecting features, that are elaborated in [27,28,32] and reviewed in Section 2.3. From these features, we can propagate a belief about the platform and mark the occurrences in the new introduced FM F. Given the platform detection output, we can either use the estimated velocity (see Section 2.3) if available or sample an assumed velocity vector $V^{F}$ that superimposes the MP displacement within one update interval.

The underlying thought is to interpret the MPs as a characteristic of the map. Our justification is its temporal stability—in normal operation mode the elevator or escalator moves with manufacture-dependent (factory-dependent) constant (absolute) velocity—and the moving constraints enforced by the car size or width between handrails. In case the platform superimposes a movement on the pedestrian, the external velocity can be perceived by the MPD (Section 2.3) and an association is made. In case the MP is out-of-order and in rest, no such velocity is detected and no association is triggered.

Our goal is to estimate the hidden states of the MPs at any time instance, which requires to compute the joint posterior $p\left(P_{0:k}U_{0:k}E_{0:k},F,M|Z_{1:k}^{U}Z_{1:k}^{F}\right)$, where $U_{k}=U_{k}^{U}+U_{k}^{F}=P_{k}-P_{k-1}$. According to the FastSLAM approach [10] the joint posterior can be factorized as follows:

(1) $$\begin{array}{c} p\left(P_{0:k}U_{0:k}E_{0:k},F,M|Z_{1:k}^{U},Z_{1:k}^{F}\right)=\underset{mapping\;\;problem}{\underset{︸}{p\left(M|P_{0:k}\right)\cdot p\left(F|P_{0:k}\right)}}\cdot \underset{localization\;\;problem}{\underset{︸}{p\left({\left\{PUE\right\}}_{0:k}|Z_{1:k}^{U},Z_{1:k}^{F}\right)}}\; \end{array}$$

which splits the estimation problem into a mapping and a localization problem. In this equation we exploited the fact that given the history of poses $P_{0:k}$, the transition map M as well as the FM F become conditionally independent from the history of step vectors $U_{0:k}$, measurements $Z_{0:k}^{U}$ and $Z_{0:k}^{F}$, and errors $E_{0:k}$.

We can express the right hand side, i.e., the localization problem in Equation (1), recursively. In accordance with the DBN structure in Figure 1 and similar to the derivation in [17], given the relationship $U_{k}=U_{k}^{U}+U_{k}^{F}=P_{k}-P_{k-1}$ and assuming that human motion is independent from using an MP the recursion formula becomes

(2) $$\begin{array}{cccc} p\left({\left\{PUE\right\}}_{0:k}|Z_{1:k}^{U},Z_{1:k}^{F}\right)\propto \; & p\left({\left\{PU\right\}}_{k}{|\left\{PU\right\}}_{0:k-1}\right) & \mathrm{pose}\mathrm{transition}\mathrm{probability} & \\ \cdot & p\left(E_{k}^{U}|E_{k-1}^{U}\right)\cdot p\left(E_{k}^{F}|E_{k-1}^{F}\right) & \mathrm{error}\mathrm{state}\mathrm{transitions} & \\ \cdot & p\left(Z_{k}^{U}|U_{k}^{U},E_{k}^{U}\right)\cdot p\left(Z_{k}^{F}|U_{k}^{F},E_{k}^{F}\right) & \mathrm{measurement}\mathrm{likelihoods} & \\ \cdot & p\left({\left\{PUE\right\}}_{0:k-1}|Z_{1:k-1}^{U},Z_{1:k-1}^{F}\right) & \mathrm{recursive}\mathrm{belief}\mathrm{propagation} & \; \end{array}$$

In our representation, we assume $Z^{U}$ to be independent from $Z^{F}$. Since the velocity is reset within each stance phase in the lower UKF, we can assume that this assumption holds. In addition, we assumed that the error $E^{U}$ is independent from $E^{F}$. Due to the fact that there are different error sources the errors are independent from each other. On the one hand, the step length and heading error resulting from the UKF represent the error sources for $E^{U}$. On the other hand, either integration of the acceleration sensor biases for calculating the elevator velocity during stance phases (or assuming a predefined velocity in escalators) and additionally heading errors of the FootSLAM algorithm (escalators) are the error sources for $E^{F}$.

In the original FootSLAM approach, the map is divided into a regular grid of adjacent uniform hexagonal prisms and the transitions stored are the transitions over hexagonal prism edges. In the proposed extension to FootSLAM, we additionally store the feature properties as additional parameters to the hexagonal prisms. In our implementation, a new feature vector is added that only needs to be stored once per hexagonal prism of the original transition map. The complexity reduced FootSLAM approach presented in [40] remains the same except that we additionally store the feature vector in each hexagonal prism.

In the following, we look closer at the first term of Equation (2), which is the pose transition probability and where the transition map as well as the FM play a role (see DBN, Figure 1). Assuming that the transition map M, as well as the FM F, are statistically independent for simplicity and that the pose and the true step ${\left\{PU\right\}}_{k}$ are only dependent on the previous pose $P_{k-1}$ and on the respective map, we marginalize this term over the transition map M and the FM F and obtain:

(3) $$\begin{array}{c} p\left({\left\{PU\right\}}_{k}{|\left\{PU\right\}}_{0:k-1}\right)\\ =\underset{I_{F}}{\underset{︸}{\int_{F}p\left({\left\{PU\right\}}_{k}|P_{k-1},F\right)\cdot p\left(F|P_{0:k-1}\right)dF}}\cdot \underset{I_{M}}{\underset{︸}{\int_{M}p\left({\left\{PU\right\}}_{k}|P_{k-1},M\right)\cdot p\left(M|P_{0:k-1}\right)dM}}\;\; \end{array}$$

where we defined $I_{F}$ and $I_{M}$ to be the integrals marginalizing over F and M, respectively. Note that both maps are not fully independent, since an MP constraints the possible transitions in the map. On the other hand, the pedestrian may also walk on the platform, so that other directions than the platform direction (e.g., up and down in an elevator) are still possible. The feature vector is restricted to contain the position, velocity and direction of the platform and not the step displacement and heading of the pedestrian. With the assumption of independent maps we can handle both maps independently and are able to calculate the weights separately.

Since the transition map is a set of hexagonal prisms $M=M_{0},M_{1},\ldots ,M_{i},\ldots ,M_{N_{h}-1}$ with $N_{h}$ hexagonal prisms, the conditioned probability of the transition map can be expressed as:

(4) $$\begin{array}{c} p\left(M|P_{0:k}\right)=\prod_{h=0}^{N_{h}-1}p\left(M_{h}|P_{0:k}\right)\; \end{array}$$

In the same way, the FM is a set of hexagonal prisms $F=F_{0},F_{1},\ldots ,F_{i},\ldots ,F_{N_{h}-1}$ with $N_{h}$ hexagonal prisms. Note that the FM consists of the same hexagonal prisms as the transition map due to the fact that we rely on the same history of poses. Therefore, the number of hexagonal prisms in the FM is equal to the number of hexagonal prisms in the transition map. The conditioned probability of the FM can be expressed as:

(5) $$\begin{array}{c} p\left(F|P_{0:k}\right)=\prod_{h=0}^{N_{h}}p\left(F_{h}|P_{0:k}\right)\; \end{array}$$

With this assumption, the integral $I_{F}$ becomes:

(6) $$\begin{array}{c} I_{F}=\int_{F_{h}}p\left({\left\{PU\right\}}_{k}|P_{k-1},F_{h}\right)\cdot p\left(F_{h}|P_{0:k-1}\right)dF_{h}\; \end{array}$$

In [16] it has been shown that the weight update for each particle *m* in the original FootSLAM approach is equal to the last particle weight times the integral: $w_{k}^{m}=w_{k-1}^{m}\cdot I_{M}^{m}$. In a similar derivation the same can be proven for the weight update including both maps—the transition map and the FM, therefore we obtain:

(7) $$\begin{array}{c} w_{k}^{m}=w_{k-1}^{m}\cdot I_{M}^{m}\cdot I_{F}^{m}\; \end{array}$$

Following this equation, we can handle the weight updates depending on the transition map and depending on the FM separately.

### 2.2. Particle Filter Implementation

**Figure 2:**
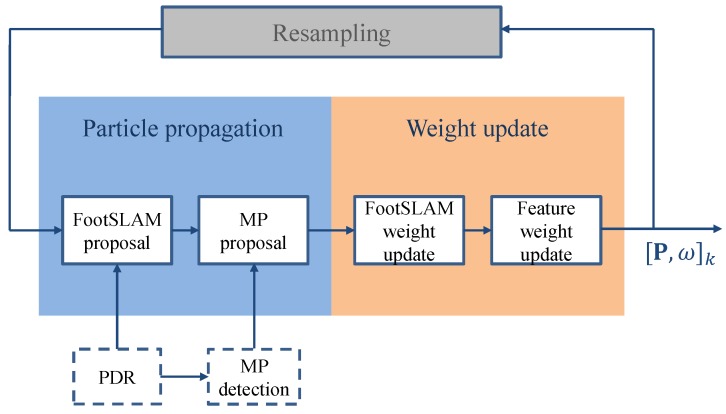
Extended particle filter implementation including the moving platform proposal function and weighting with the feature map.

The FootSLAM framework builds on a RBPF to simultaneously address the mapping and localization problem, and estimates the full posterior of the DBN in Figure 1. It is implemented as a likelihood particle filter, according to [41], which samples from a likelihood-based density conditioned on the most recent measurement. Figure 2 shows the extended particle filter implementation including the MP proposal function and weighting with the FM. In the following, the proposal functions and the particle weighting process are described.

#### 2.2.1. Proposing Particle State Propagation

The particle position is intuitively propagated twice, accounting for the superposition of stepped locomotion and external displacement from MPs. For any particle where an MP is detected we sample a second time based on the feature.

In the original FootSLAM algorithm the particle *m* is propagated by sampling from the FootSLAM proposal density $p\left(E_{k}|E_{k-1}^{m}\right)\cdot p\left(U_{k}|Z_{k}^{U},E_{k}^{m}\right)$, which is conditioned on the step vector measurement $Z_{k}^{U}$ provided by the underlying PDR-system [16,19].

The actual step taken consists, in our case, of two components $U_{k}=U_{k}^{U}+U_{k}^{F}$, which are statistically independent. With this, the proposal function can be decomposed into two components, which are described next.

##### FootSLAM Proposal Density

Each particle *m* is proposed from the proposal density function only dependent on the step measurements and step measurement errors: $p\left(E_{k}^{U}|E_{k-1}^{U,m}\right)\cdot p\left(U_{k}^{U}|Z_{k}^{U},E_{k}^{U,m}\right)$.

In the error model different kinds of errors are intuitively considered: additive white translational noise, white noise on the heading change and coloured heading noise [16,19] based on a random walk process of order 1. Additionally, the error of the z-component of the odometry is modeled by an auto-regressive integrated moving average (ARIMA) process [23]. The pose is proposed based on the incoming measurements and finally the error is added to the proposed pose (position and heading).

##### Moving Platform Proposal Density

Similarly to the FootSLAM proposal process in case of an MP event each particle *m* is additionally propagated from the following proposal density function: $p\left(E_{k}^{F}|E_{k-1}^{F,m}\right)\cdot p\left(U_{k}^{F}|Z_{k}^{F},E_{k}^{F,m}\right)$.

Different MPs transport the user with varying velocities and inclinations. Therefore, in addition to the estimated velocity we used legal regulations for elevator and escalator construction to restrict the range for the angle of inclination and the speed of the MP. For instance, in Europe, the angle of inclination is restricted to be less or equal to $35^{{}^{\circ}}$ and the nominal speed is restricted to be less than or equal to 0.75 m/s for escalators, whereas the angle of inclination of elevators is $0^{{}^{\circ}}/180^{{}^{\circ}}$ and the nominal speed of elevators is defined to be between 0.5 m/s and 4 m/s [42].

For the velocity of elevators, we directly used the estimated velocity and considered an additive white noise error for it. In case of escalators, we might miss the beginning of the acceleration phase during the gait phases due to perturbing accelerations while stepping on the platform. Therefore, it turned out to be better to propose the platform motion vector from a prior distribution that takes into account velocities and inclinations from escalators usually built in Germany.

The platform heading for elevators is assumed to be $0^{{}^{\circ}}$ or $180^{{}^{\circ}}$ depending on the sign of the z-component of the velocity of the platform. The platform heading for escalators is drawn from a normal distribution with mean corresponding to the previous step heading and a small variance. This is based on following intuitive observation: a pedestrian has to walk onto the escalator on her own. The entry for an escalator is guided by the two handrails in parallel to the direction of movement. Consequently, the last step with a horizontal distance of more than the length of a foot, is more or less aligned with the escalator heading.

#### 2.2.2. Computing Particle Weights

##### FootSLAM Weight Update

The displacement likelihoods are assigned by the step direction probability that is derived from the hexagonal prism map M. A detailed description can be found in [17].

##### Feature Weight Update

For computing the feature weight update, a new feature vector $F_{h}$ is added to each particle’s transition map. The feature vector consists of $N_{F}$ different features where each value represents a feature counter for each feature, i.e., the number of times the feature is recognized. The FM F stores for each hexagonal prism of our map the feature counters $F_{h}$ in order to obtain an estimation of the FM. In our case we used the following $N_{F}=3$ features: Escalator,up,Escalator,down,Elevator. Adding new features or changing the features is possible, e.g., the feature stairs is foreseen for future work. Since the location of the elevator in our hexagonal prism map usually does not depend on the direction, we have added only one value for being in an elevator. For the escalator, we distinguish between the locations for an escalator going up and an escalator going down because the locations of the escalator mostly differ depending on the direction, and the differentiation will make it more reliable in terms of positioning accuracy assuming enough distance between both locations. This includes also escalators that do not differ in the location for going up or down due to the fact that they are able to change the direction. Then, both features are counted in the same hexagonal prism.

Similar to the approach that $p(M_{h}|P_{0:k-1})$ in FootSLAM follows a Dirichlet distribution [43], we assume that $p(F_{h}|P_{0:k-1})$ follows a Dirichlet distribution. Hereby, we assume that learning the FM is based on Bayesian learning of multinomial distributions using Dirichlet priors [44].

In the FM, we distinguish between $N_{F}$ different features at each hexagonal prism *h*. Each time the hexagonal prism is crossed we count the number of times the feature is recognized. The Dirichlet distribution represents the belief that the probabilities of the $N_{F}$ events, i.e., features are $\left\{p\left(F_{h}^{0}|P_{0:k-1}\right),p\left(F_{h}^{1}|P_{0:k-1}\right),\ldots ,p\left(F_{h}^{N_{F}-1}|P_{0:k-1}\right))\right\}$ under the assumption that each of the events—in our case each of the features—was observed $u_{i}$ times, where $u_{i}$ represent the so called parameters of the Dirichlet distribution that are positive real numbers.

The feature counters represent the counters of the occurrences of each feature. An initial prior distribution is assumed to be uniform and of low value. The $N_{F}$ parameters $u_{h}^{i}$ of the Dirichlet distribution are therefore the feature counters $f_{h}^{i}$ plus an additional prior value $\alpha_{f_{h}}^{i}$ and the parameter vector $\vec{u_{h}}$ can be written as:

$${\vec{u}}_{h}={\vec{f}}_{h}+{\vec{\alpha }}_{fh}=\left\{f_{h}^{i}+\alpha_{f_{h}}^{i}\right\}\;\forall \;i=0,\ldots ,N_{F}-1\;$$

with

$$u_{h}=f_{h}+\alpha_{f_{h}}=\sum_{i=0}^{N_{F}-1}\left(f_{h}^{i}+\alpha_{f_{h}}^{i}\right)\;$$

The Dirichlet distribution can be written as:

(8) $$\begin{array}{c} D(p\left(F_{h}^{0}|P_{0:k-1}\right),\ldots ,p\left(F_{h}^{N_{F}-1}|P_{0:k-1}\right);f_{h}^{0}+\alpha_{f_{h}}^{0},\ldots ,f_{h}^{N_{F}-1}+\alpha_{f_{h}}^{N_{F}-1})\\ =\frac{1}{B({\vec{f}}_{h}+{\vec{\alpha }}_{fh})}\prod_{i=0}^{N_{F}-1}p{\left(F_{h}^{0}|P_{0:k-1}\right)}^{f_{h}+\alpha_{f_{h}}-1}\;, \end{array}$$

where

$$B\left(\vec{u}\right)=\frac{\prod_{i=0}^{N_{F}-1}\Gamma \left(u_{i}\right)}{\Gamma (\sum_{i=0}^{N_{F}-1}u_{i})}\;$$

is the Beta function and $\Gamma \left(x\right)=\int_{0}^{\infty }e^{-t}t^{x-1}dt\;$ is the gamma function.

In order to obtain the weights for each particle, we have to calculate the integral $I_{F}$ of Equation (6) with the assumption that $p(F_{h}|P_{0:k-1})$ follows a Dirichlet distribution. Since the probability of the feature $F_{h}^{i}$ is proportional to the probability $p\left({\left\{PU\right\}}_{k}|P_{k-1},F_{h}\right)$ the integral $I_{F}$ can be solved to [44]:

(9) $$\begin{array}{c} I_{F}^{m}\propto {\left\{\frac{f_{h}^{i}+\alpha_{f_{h}}^{i}}{f_{h}+\alpha_{f_{h}}}\right\}}^{m}\; \end{array}$$

With this equation, we are able to calculate the weights of the particles by multiplying the original FootSLAM weights with the new feature weighting function. The prior values are set to $\alpha_{f_{h}}^{i}=0.8$ in order to be consistent with the FootSLAM approach and not to favor particles because of their feature vector at the beginning. It should be noted that weighting with the FM is only performed when a feature is detected.

### 2.3. Moving Platform Detection

During the static phases of the sensor the acceleration phase of an MP can be detected via integrating and thresholding the velocity. However, small bias mismatches will accumulate in the velocity estimate leading to detection failures and velocity estimation errors. Therefore, a new acceleration phase tracking method based on the estimation of the acceleration during the phases when the sensor is declared as static was proposed in [28]. It allows to correct the velocity estimate during the constant velocity phase of the MPs.

Since the acceleration phase tracking is very accurate in terms of timing and detection, in this work we solely used the acceleration phase tracking and velocity estimation described in [28]. The classification of the platform is performed by comparing the maximum velocity measured in the acceleration phase. The maximum velocity of an elevator is much higher than that of an escalator, therefore, this classification is reliable. At the beginning of the platform before the calculated velocity reaches the maximum in the acceleration phase we assume both possibilities, escalators or elevator with equal probability, using the estimated speed. Combinations with other techniques such as proper feature selection and applying classifier algorithms from the area of activity recognition [32] or the use of other sensors like magnetometers and barometer [27] are foreseen for future work.

## 3. Experimental Results

### 3.1. Experimental Settings

**Figure 3:**
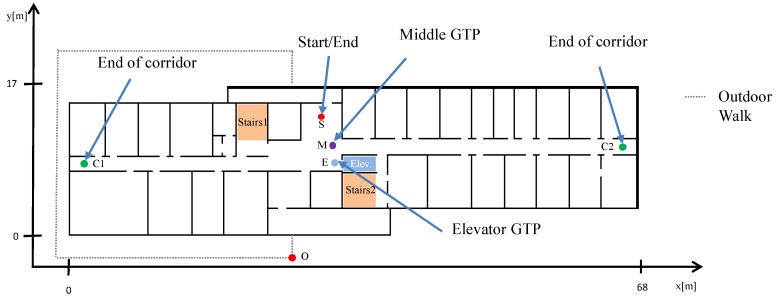
Building layout of our office building at Oberpfaffenhofen. The GTPs, the stairs and the elevator are marked in different colors. The dotted line represents an outdoor transition.

To verify the proposed approach, we collected different walks in two different environments with a sensor mounted on the foot of the pedestrian. In order to provide a quality comparison we crossed predefined ground truth points (GTPs) available in each floor level of our office environment including floor changes between five different floor levels. The positions of the GTPs were measured with a laser distance measurer (LDM, millimtere accuracy) and a tape measurer (sub centimeter range accuracy). The GTPs serve as a reference. Figure 3 shows the different GTP locations in one floor. The same GTP locations are used in the other floor levels. Two pedestrian—referred to as (1) and (2)—collected data during four walks with different kinds of floor changes using the elevator and the stairs cases. Whenever the pedestrian crossed the GTP, the pedestrian stopped for 2–3 s at this point and the time of passing the GTP is logged. The pedestrians walked (37,9,10,13) and (19,9,10,15) min with distances of (1540,160,335,325) m and (773,159,314,271) m as estimated by FootSLAM for walks 1–4 pedestrian (1)/(2), respectively, wearing an XSens MTw IMU mounted on the foot during the walk in our office environment.

The varying types of floor level changes of the four walks in our office building are summarized in Table 1. These walks were performed twice by two different pedestrians. The first walk of one pedestrian was additionally repeated twice in order to provide one long walk of 37 min. The GTPs and stair cases used in these walks are given in Table 2 and the chronological order of the GTPs used is given in Table 3. In the third walk, the pedestrian started outdoors, went in the building passing two different floor levels (floor changes by the elevator and stairs), left the building again and walked around the building before he entered the building at the opposite side of the building (see Figure 3) in order to test the sensor in a different environment (magnetic and air pressure changes, uneven ground). He took the elevator one last time to the second floor.

In addition to the walks performed in our office building, three same walks were performed in a shopping center, where the pedestrian used the escalator, the stair cases, and the elevator as described in Table 4. The pedestrian walked 4.6–4.8 min with distances of 215–224 m wearing an XSens MTX IMU during the walk in the shopping center. Unfortunately, in these walks no GTPs were available in order to provide a quality measure of the walks. Here, we compare the estimated maps in order to provide a comparison of the results. These three walks show the convergence behavior when using elevators and escalators as features.

The data processed by the lower UKF [6] are used as input for the 3D-FootSLAM algorithm [17,23]. Beside this, we used the MPD algorithm described in [28]. The hexagon radius of the hexagonal prisms was chosen to be r=0.5 m and we used six hexagon prisms between each floor level. The number of particles of the RBPF was $N_{p}=$ 10,000. Starting conditions (initial heading and position) are assumed to be known. 3D-FootSLAM needs as input the height between the floor levels which was 3.5 m for the office building and 6 m for the shopping mall. For the shopping mall we estimated the height between the floors to be the height of the first floor level change using stairs. In case of our office building the xy-error (2D error) and z-error (height error) are calculated as the Euclidean distance to the GTPs points, respectively.

### 3.2. Experimental Results

**Figure 4:**
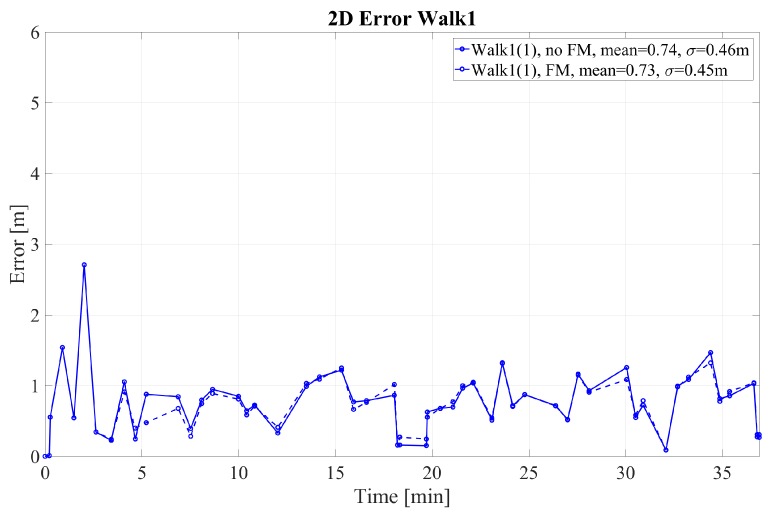
2D error at the GTPs for walk 1 performed by pedestrian (1). The results are shown with and without weighting with the feature map (FM).

**Figure 5:**
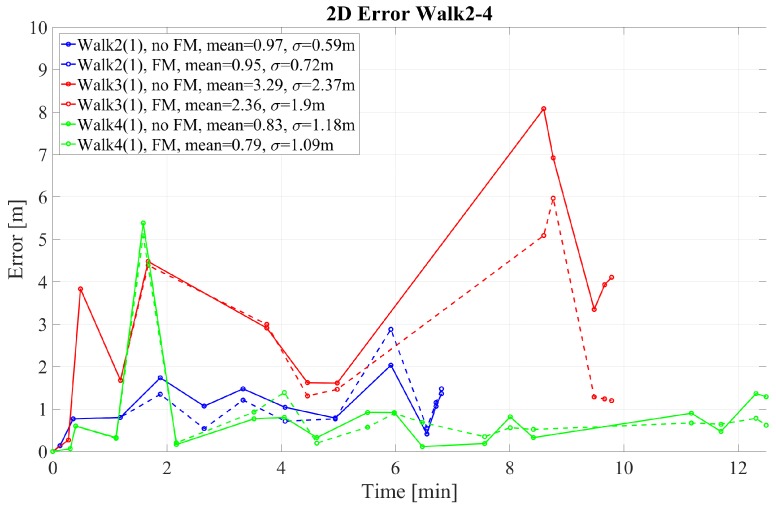
2D error at the GTPs for walks 2–4 performed by pedestrian (1). The results are shown with and without weighting with the feature map (FM).

**Figure 6:**
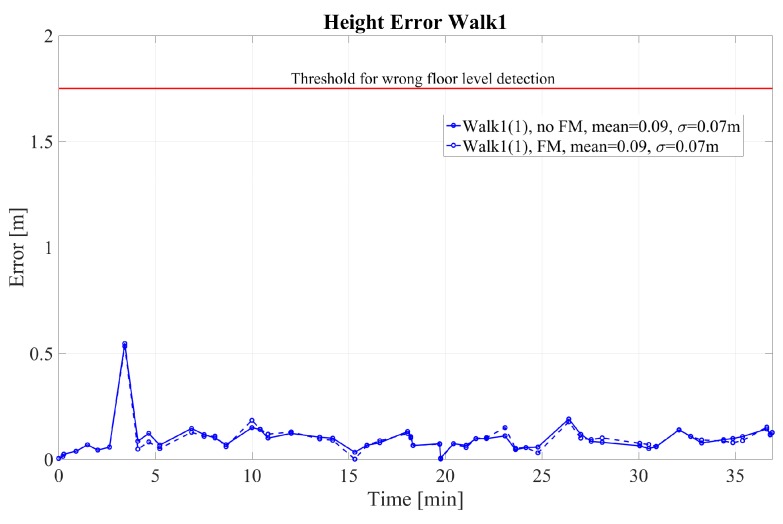
Height error at the GTPs for walk 1 performed by pedestrian (1). The results are shown with and without weighting with the feature map (FM).

**Figure 7:**
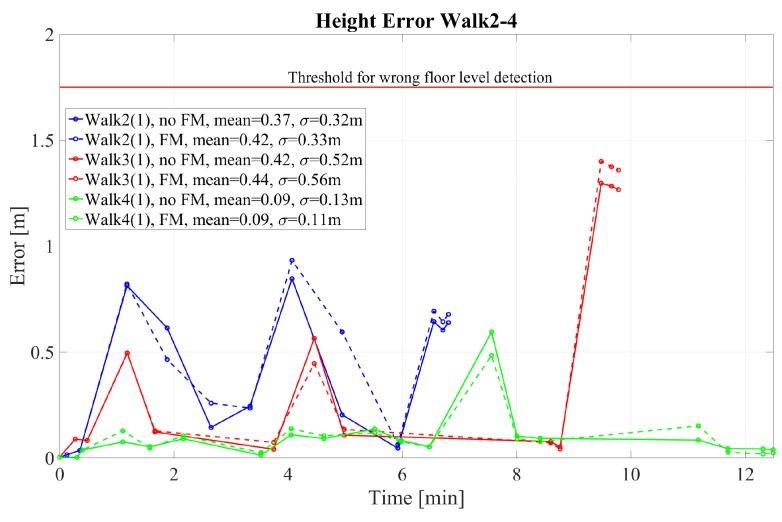
Height error at the GTPs for walks 2–4 performed by pedestrian (1). The results are shown with and without weighting with the feature map (FM).

The 2D error for walks 1–4 performed by pedestrian (1) is given in Figure 4 and Figure 5 for walk 1 and walks 2–4, respectively. The corresponding height error is given in Figure 6 and Figure 7. In the following, we mark the results with a (1) and (2), respectively, following the walk name to distinguish the walks of the two different pedestrians. In the Figure 4, Figure 5, Figure 6 and Figure 7, the results are real time errors. They are given for using the new elevator proposal function only, i.e., not weighting with the FM (no FM), and for using additionally the FM (FM).

The results show that for the very long walk 1(1) a high accuracy can be reached. The mean 2D error is below 1 m when calculating the Euclidean distance at the GTPs. In addition, the mean height error is only 0.09 m. In this walk, long straight segments were passed, where FootSLAM was able to converge to the correct 2D map and the correct heights. At the beginning FootSLAM searches for the best direction of the path until convergence is reached. This is reflected in the high 2D error peak at the beginning of the walk. All floor level changes performed via the elevator are handled correctly also for different amplitudes of floor level changes. In addition, during one elevator ride (last elevator ride of walk 1(1)) the elevator was going down before it went up to the requested floor level because other people went in and chose the wrong floor level (real live situation). This floor level change was also handled without errors.

The second walk in our office building was a more challenging walk because long, straight segments were avoided. The results for the 2D error and the height error for the second walk in our office building are given in Figure 5 and Figure 7, respectively. They show that it is possible to detect all different floor levels without performing long walks. The 2D error is below one meter and the height error is on average below 0.5 m which makes us confident not to confuse floor levels. The threshold would be in our case 1.75 m which is half of the floor level height. It should be noted that after the elevator we directly passed the middle GTP, where only a short way from the elevator leads to it. We observed that after the middle GTP the map converged to the correct level again after walking a while on that level. Therefore, the height error is sometimes higher at the middle GTP. Due to the fact that the elevator velocity is sometimes not correctly estimated, directly after the elevator the height might be under or overestimated. This causes height errors which will be corrected afterward when walking on the same floor due to preferring equal floor level heights inside the FootSLAM algorithm.

The 2D error of walk 3 in Figure 5 suggests that comparable long walks outdoors providing no loop closures and without receiving GNSS are also challenging. In general, FootSLAM converges faster when using the FM, which shows lower 2D and height errors (see end of walk 3, Figure 5). Furthermore, without the FM it failed to converge in walk 3 (wrong elevator position). Moreover, at the very end of the walk the pedestrian left the elevator and walked to the end point in the second floor. This path was again very short so that the height was not yet corrected (see Figure 7).

Finally, Figure 5 and Figure 7 show the errors for walk 4 of pedestrian (1). In this walk the pedestrian took the elevator four times with different positions inside the elevator (at the four corners of the elevator). Despite the different positions the performance of the walk was below one meter. The height error arose once to ≈0.5 m when walking shortly from the elevator to the middle GTP. These peaks occurred again when the elevator velocity was uncertain, e.g., if the beginning of the elevator was detected late. After a while the estimator returned to the correct height again. It should be noted that the positions in the elevator were distributed over two hexagonal prisms inside the FootSLAM map. Since the radius of the underlying hexagons was 0.5 m and the elevator car is of rectangular form where each two corners are very close to each other, the positions do not differ in terms of hexagon prisms.

Since the convergence behavior of the particle filter depends also on the seed of some random generators, we used different seeds and simulated the walks ten times in order to verify the results. Table 5 shows the mean 2D error and the mean height error for the four walks of the two pedestrians. In addition to the real time performance the performance using the best estimated map as prior map is given in the table. The offline performance (i.e., using the best prior map as input) is considered for approximating the performance of the best particle since we do not store whole paths of all particles in the FootSLAM states. Due to the fact that FootSLAM is only directed to the prior map but still estimates the path including searching the direction of the walk, using the best particle map as prior only provides a rough estimate of the path of the final best map. From the table we can see that for walks 1, 2 and 4 similar performance can be reached with and without the FM. The 2D error performance was below or close to 1 m except for walk 3. Especially for walk 3(1), the converged map was sometimes slightly rotated due to a high heading drift from the beginning on, which is reflected in the 2D error results. The correct floor number though is in all cases except one correctly estimated, which is also reflected in the small height errors of Table 5. In the case of the failure in floor level estimation, the velocity estimate was erroneous, and the walk was very short after the elevator and ended before FootSLAM was able to detect a loop closure (end of walk 4(2)). Here, the particles are distributed over different hexagonal prisms which may include different levels. In many cases the height accuracy was below 0.1 m, and in some cases the mean height error was 0.5 m. For the walks with such inaccuracies it was observed that FootSLAM returns in all cases (except one) after a while to the correct height level. In future work we want to ensure the numbers of floors to be changed with the inspection of the duration of constant velocity phase during the elevator ride. If the duration of the the constant velocity phase for one floor level change is known, the duration of the constant velocity phase shows clearly the number of floors to be changed. The duration of a floor level change can be learned during the walk for a particular elevator.

Since FootSLAM is robust in 3D environments when having loop closures—and the elevator serves as a loop closure with same upper and lower face transitions—the advantages of using the FM cannot directly be seen by those walks. For walk 3, however, for both pedestrians the performance was more accurate when using the FM. Especially after long phases without any loop closure the FM helped to converge faster and position accuracy is increased.

**Figure 8:**
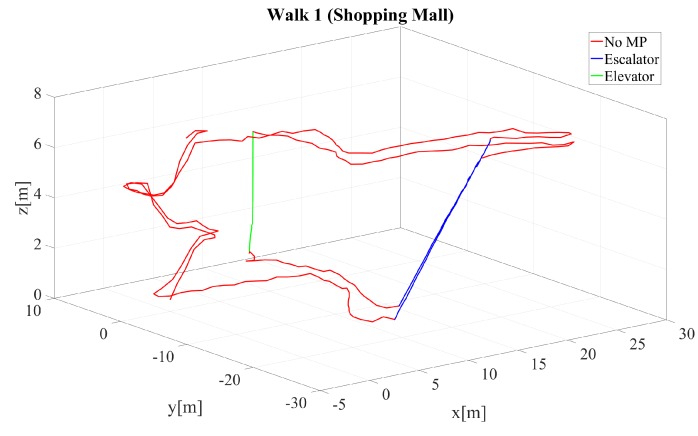
Trajectory of walk 1 in the shopping mall. The elevator is marked in green and the escalator in blue.

**Figure 9:**
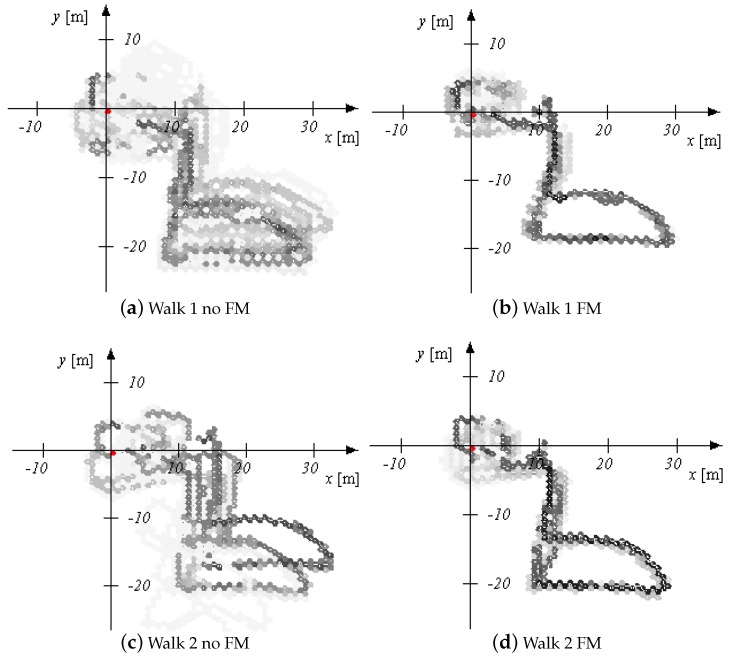

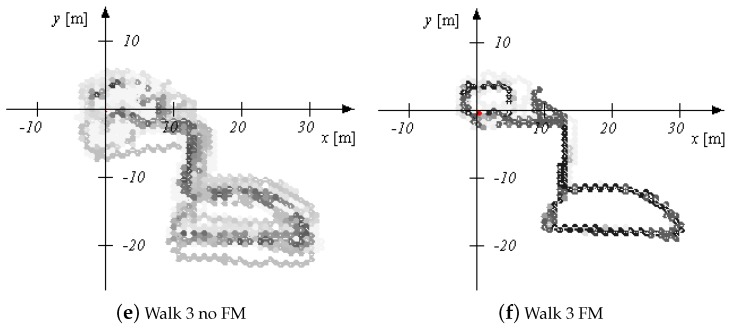
Accumulated posterior maps of walks 1–3 in the shopping mall. The aggregated posterior maps are given for not using the feature map (no FM) and for using the feature map (FM). The posterior for using the FM are more reliable than the posterior maps for not using the FM.

Finally, the resulting trajectory of walk 1 in the shopping mall is given in Figure 8. The escalator is marked in blue, whereas the elevator is marked in green. One can see that the movements of the MPs are reproduced, the two different floor levels are clearly distinguished, and the heading drift is corrected. In this walk, the escalator is used twice where the FM comes into play. In order to compare the convergence behavior of the three walks in the shopping mall, we depicted the final aggregated posterior map, i.e., the sum of all weighted particle maps, in Figure 9 for the three walks not using and using the FM, respectively. This walk is also challenging due to the fact that the shopping mall contains wide areas without wall constraints and the pedestrian took the elevator only once (no loop closure). The only loop closure based on features was the second escalator ride which was very late. Figure 9 shows that the aggregated maps are more uncertain for the walks when not using the FM and more certain when using the FM. This leads us to the conclusion that it is advantageous to use the FM for a more reliable position and map estimation.

## 4. Conclusions

Moving platforms are not yet handled in many indoor navigation systems based solely on PDR. In this paper, we provided an approach for integrating moving platforms in the so called FootSLAM algorithm. A new proposal function is introduced when the pedestrian is on the platform. It is based on the velocity estimate given by the moving platform detector. In addition, a feature indicator is included in the FootSLAM map representation and a weighting function is added based on the feature indicator. It has been shown that 3D FootSLAM robustly estimated the height of each floor level. The system was able to handle varying floor level changes in multistory buildings. Finally, including moving platforms as features in the FootSLAM map is beneficial especially in cases where the loop closure is very far and it turned out that the resulting aggregated posterior map is more reliable.

Walking on escalators is not yet considered in the analysis and is foreseen for future research. Here, the difficulty is to reliably detect the escalators and to estimate the displacement due to the moving platform correctly. In addition, a confidence metric for the detection, classification, and velocity estimation of moving platforms would be desirable in order to provide a probability of the goodness of the detection, classification, and velocity estimate for the Bayesian estimator.

While the system presented in the paper is solely based on foot-mounted PDR, which is especially interesting for professional use cases like fire fighters rescuing or policemen supervision, future work should involve the migration to other or unrestricted sensor locations and the integration of further combined sensor modalities like WiFi, RFID, or prior maps. 

## Figures and Tables

**Table 1:** Floor changes of the four different walks inside our office building.

Walk 1		Walk 2		Walk 3		Walk 4	
Floor Level	Next Floor	Floor Level	Next Floor	Floor Level	Next Floor	Floor Level	Next Floor
	Change		Change		Change		Change
2	elev. up	2	elev. down	0	elev. up	2	elev. down
3	stairs down	1	stairs up	2	elev. up	1	elev. up
1	elev. up	2	elev. up	3	stairs down	3	elev. down
4	stairs down	3	stairs down	0	elev. up	0	elev. up
2	elev. down	2	elev. up	2		4	elev. down
0	stairs up	4	elev. down				
2		2	stairs down				
		0	elev. up				
		2					

**Table 2:** GTPs and stairs used in the four walks inside our office building. S is the start/end, C the end of corridor, M the middle, and E the elevator GTP.

	GTP S	GTP C	GTP M	GTP E	Stairs 1	Stairs 2
Walk 1	x	1,2	x	x		x
Walk 2	x		x	x	x	x
Walk 3	x	1	x	x	x	
Walk 4	x	1	x	x		

**Table 3:** Chronological order of the GTPs used in the four walks inside our office building. The first letter is the level (0–4), the second letters are the GTPs: S (start/end), M (middle), E (elevator), C1 (end of corridor 1), C2 (end of corridor2). O is the outdoor GTP in walk 3.

	GTPs
Walk 1	2S, 2M, 2C2, 2M, 2C1, 2E, 3M, 3C1, 3M, 3C2, 1C2, 1M, 1C1, 1E
	4C1, 4M, 4C2, 2M, 2C1, 2E, 0C1, 0M, 0C2, 2M, 2S
Walk 2	2S, 2E, 1M, 2E, 3M, 2E, 4M, 4E, 2M, 0M, 0E, 2M, 2S
Walk 3	O, 0E, 2M, 2C1, 2E, 3M, 3C1, 0M, 0E, 2S
Walk 4	2S, 2E, 1M, 1C1, 1E, 3M, 3C1, 3E, 0M, 0C1, 0E, 4M, 4C1, 4E, 2M, 2C1, 2S

**Table 4:** Floor changes of the three different walks in the shopping mall.

Walks 1–3	
Floor Level	Next Floor Change
0	stairs up
1	escalator down
0	elevator up
1	escalator down
0	stairs up

**Table 5:** Mean 2D error (2DE) and mean height error (HE) for walks 1–4 in our office building not using the feature map (no FM) and using the feature map (FM). The real time error (RTME) is depicted first following by the error performance using the best map as prior map (PM).

	Walks 1–4(1)				Walks 1–4(2)			
	No FM		FM		No FM		FM	
	2DE (m)	HE (m)	2DE (m)	HE (m)	2DE (m)	HE (m)	2DE (m)	HE (m)
Walk 1 RTME	0.74	0.09	0.73	0.11	0.88	0.09	0.88	0.09
Walk 2 RTME	1.16	0.4	1.26	0.5	0.7	0.06	0.63	0.07
Walk 3 RTME	3.41	0.45	2.91	0.45	2.3	0.12	1.84	0.13
Walk 4 RTME	0.84	0.09	0.86	0.1	0.84	0.55	0.88	0.64
Walk 1 PM	0.73	0.08	0.74	0.09	0.75	0.08	0.83	0.09
Walk 2 PM	1.09	0.38	1.15	0.47	0.66	0.05	0.6	0.06
Walk 3 PM	2.33	0.41	2.12	0.41	1.54	0.1	0.84	0.09
Walk 4 PM	0.59	0.08	0.56	0.09	0.69	0.53	0.74	0.62

## Abbreviations

The following abbreviations are used in this manuscript:

DLR	German Aerospace Center
DLR-KN	Institute of Communication and Navigation at the german aerospace center
IMU	inertial measurement unitt
INS	inertial navigation system
PDR	pedestrian dead-reckoning
GNSS	global navigation satellite system
UKF	unscented Kalman filter
MEMS	micro-mechanical systems
SHS	step-and-heading system
PF	particle filter
RBPF	Rao-Blackwellized particle filter
AMM	activity map matching
ZUPT	zero velocity update
ZARU	Zero Angular Rate Updat
MARU	Magnetic Angular Rate Update
HMM	hidden Markov model
ISA	International Standard Atmosphere—a norm for an atmospheric model used by the US Air Force
baro-IMU	inertial measurement unit equipped with a barometric pressure sensor
MPD	moving platform detection
SIR	Sampling Importance Resampling
PDF	probability density function
CDF	cumulative distribution function
DBN	dynamic Bayesian network
SLAM	simultaneous localization and mapping
MP	moving platform
FM	feature map
MP(MPs)	moving platforms
IMU(IMUs)	inertial measurement units
